# Brachial pulse pressure is associated with the presence and extent of coronary artery disease in stable angina patients: a cross-sectional study

**DOI:** 10.1186/s12872-020-01416-1

**Published:** 2020-03-20

**Authors:** Jin Li, Yangpei Peng, Kangting Ji

**Affiliations:** Department of Cardiology, The Second Affiliated Hospital of Wenzhou Medical University, Wenzhou, 325000 Zhejiang China

**Keywords:** Brachial pulse pressure, Coronary artery disease, Stable angina

## Abstract

**Background:**

Previous epidemiological evidence has identified many risk factors for coronary artery disease (CAD). Pulse pressure (PP) was reported to be associated with CAD. However, more attention was paid to aortic PP than to brachial PP. This cross-sectional study aimed to investigate the direct relationship between brachial PP and the presence and extent of CAD in stable angina patients.

**Methods:**

We recruited a total of 1118 consecutive patients with stable chest pain suspected of CAD. After screening with exclusion criteria, 654 patients were finally included in our study. Every patient underwent both blood pressure measurement and selective coronary angiography. Univariate and multivariate analysis were performed to analyze the association between PP and the presence and extent of CAD.

**Results:**

This study revealed that brachial PP was an independent correlate of multivessel CAD. In multivariate generalized linear regression model, increasing brachial PP (per 1 mmHg) were associated with the increased number of diseased vessels (β = 0.01, SE = 0.00, *P* < 0.0001). Binary logistic regression analysis further confirmed this association. The risk of multivessel CAD increased significantly in patients with brachial PP ≥ 60 mmHg (OR = 1.69, 95% CI = 1.14–2.48, *P* = 0.0084) and as per 1 mmHg increased in brachial PP (OR = 1.02, 95% CI = 1.01–1.03, *P* = 0.0002), independent of age, gender, body mass index (BMI), smoking, diabetes, hypercholesterolemia and creatinine (Cr). This association was still of statistical significance in subgroup analysis of hypertension and diabetes.

**Conclusion:**

Increasing brachial PP was significantly and independently associated with increased risk of multivessel coronary disease in stable angina patients. The association of brachial PP with CAD was more pronounced in hypertension group than in non-hypertension one.

## Background

Coronary artery disease (CAD) is the leading cause of death worldwide [1, 2]. Given the high morbidity and mortality of CAD, its early diagnosis and prevention have attracted the attention of medical workers. Epidemiological evidence has identified many risk factors for CAD. Pulse pressure (PP), defined as the difference between systolic and diastolic blood pressure (SBP and DBP), has also been declared to be associated with CAD. Increased PP was shown to be related to arterial stiffness [3–5] and adverse cardiovascular events [6–9]. Several studies have investigated the association between PP and the extent of CAD in patients undergoing invasive coronary angiography (CAG). Their main focus, however, was aortic pressure, not brachial pressure [10–13]. Brachial PP, as a non-invasive and easily available indicator, can be better applied in clinical practice. Therefore, in this cross-sectional study, we explored the relationship between brachial PP and the presence and extent of coronary artery disease in the stable angina patients.

## Methods

### Study population

**Fig. 1:**
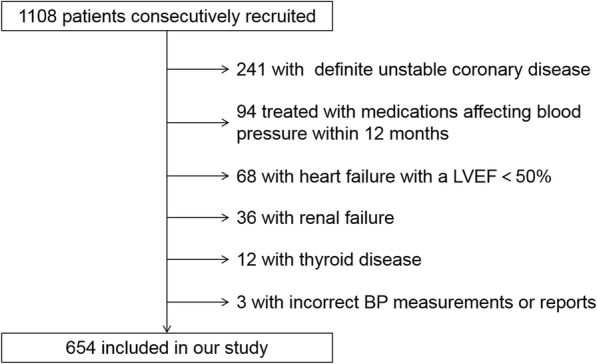
Flow chart of the population included in the study

From December 2012 to February 2014, we consecutively recruited 1108 patients with stable chest pain suspected of CAD undergoing selective CAG. We excluded those with unstable angina, non-ST segment elevation myocardial infarction, ST-segment elevation myocardial infarction, previously coronary angiographically confirmed CAD or a history of revascularization (241). Patients with any of the following conditions were also excluded: treated with medications affecting blood pressure (BP) within 12 months (eg, calcium channel blockers, β-blockers, angiotensin-converting enzyme inhibitors, etc.) (94); a history of heart failure with a left ventricular ejection fraction (LVEF) less than 50% (68); renal failure (36), thyroid diseases (12), or incorrect BP measurements or reports (3). A total of 654 patients were finally included in our study (Fig. 1).

To obtain the required information, every individual received standardized interviews and basic checks prior to the CAG. Body mass index (BMI) was calculated by dividing weight (kg) by height (m^2^). Smoking status depended on self-report. Diabetes was defined as fasting blood glucose≥7.1 mmol/L and/or random blood glucose≥11.1 mmol/L and/or receiving hypoglycemic treatment. Hypercholesterolemia was defined as total cholesterol≥5.72 mmol/L and/or low-density lipoprotein cholesterol≥3.64 mmol/L and/or receiving lipid-lowering treatment. Serum creatinine (Cr) was determined by the biochemical analyzer and expressed in μmol/L. Hypertension was defined on the basis of previous diagnoses and also the two BP measurements taken during the study (SBP ≥ 140 mmHg and/or DBP ≥ 90 mmHg).

This study conformed to the Declaration of Helsinki and was approved by the institutional review committee of Wenzhou Medical University. We obtained the written informed consent of each enrolled patient.

### Coronary angiography

All patients underwent CAG examination by standard Judkin’ s techniques. Visual analyses were applied to assess the percentage of coronary lumen diameter stenosis. CAD was defined as a 50% luminal diameter stenosis of a major epicardial artery or the left main coronary artery. Multivessel CAD was defined as a 50% luminal diameter stenosis in at least two major epicardial vessels or the left main coronary artery.

The coronary angiograms were reviewed by two experienced cardiologists who were blinded to the results of brachial PP.

### Brachial PP calculation

PP was defined as the difference between SBP and DBP. Brachial BP was measured on the basis of internationally accepted standard measurements. The brachial BP was measured within 24 h on patient’s first admission. We used the calibrated mercury sphygmomanometer and the strips manchet of right size. Before measuring BP, smoking or drinking coffee was prohibited within 30 min. Also, patients should empty the bladder and sit quietly for at least 5 min. When measuring BP, patients were asked in sitting position with bare upper arm at heart level. If peripheral vascular disease was suspected, the BP was measured both on the left and right upper arm and the higher reading was recorded. BP was expressed in millimeter of mercury (mmHg). Measurements were repeated 1 to 2 min apart and the average of two readings was recorded. If two readings of SBP or DBP differed by more than 5 mmHg, the BP then was remeasured and the average of the three readings was recorded.

### Statistical analysis

Continuous data were expressed as mean and standard deviation (SD), while discrete data as numbers and percentages. Continuous data were compared by the variance analysis or the Kruskal-Wallis test, while discrete data by the chi-square test [14] or Fisher’s exact test [15].

Univariate and multivariate generalized linear regression analyses were used to determine the relationship between brachial PP and the number of diseased vessels. Confounders were selected on the basis of their relevance to the outcome or the presence of more than 10% mutations in effect estimate or *P* values< 0.1. Multivariate logistic regression analyses were applied to investigate the relationship between brachial PP and the multivessel CAD. Subgroup analyses were performed to further explore the relationship between brachial PP and CAD.

For missing of covariates (BMI was missing 93; Cr was missing 86), we used multiple multivariate imputations. Our purpose was to maximize statistical power and minimize bias caused by excluding covariates of missing data in data analysis. In addition, we used sensitivity analysis to identify whether created complete data had significant difference from pre-imputation data. Our findings demonstrated that created complete data showed no significant difference from raw data.

We regarded a *P* value < 0.05 as of statistical significance. EmpowerStats version 2.17.8 (http://www.empowerstats.com/cn/) and R software (http://www.R-project.org) were used for statistical analysis in our study.

## Results

### Baseline characteristics

Patient demographics are presented in Table 1. Among the 654 subjects included, 374 were classified as CAD and 280 as non-CAD. The patients were stratified according to the number of diseased coronary vessels (0, 1, 2, and 3). And patients with 0-vessel disease are the controls. There were significant differences for age, gender, smoking, diabetes, and Cr across the four groups, while no significant differences for BMI and hypercholesterolemia. For BP, significant differences were observed in hypertension, SBP and PP, but not in DBP, as the number of diseased coronary vessels increased.

**Table 1:** Demographic Characteristics of the Patients^a^

Parameters	Non-CAD	CAD			*P*-value
	*n* = 280	One-vessel (*n* = 171)	Two-vessel (*n* = 87)	Three-vessel (*n* = 116)	
Age, year	61.68 ± 10.11	65.88 ± 9.77	66.76 ± 7.42	68.94 ± 7.85	< 0.001
Male, no. (%)	128 (45.71)	93 (54.39)	53 (60.92)	77 (66.38)	< 0.001
BMI, kg/m^2^	24.16 ± 3.30	24.42 ± 3.74	24.35 ± 2.81	23.97 ± 3.00	0.679
Smoking, no. (%)	79 (28.21)	65 (38.01)	39 (44.83)	57 (49.14)	< 0.001
Diabetes, no. (%)	47 (16.79)	37 (21.64)	26 (29.89)	43 (37.07)	< 0.001
Hypercholesterolemia, no. (%)	89 (31.79)	64 (37.43)	40 (45.98)	49 (42.24)	0.054
Cr, μmol/L	75.26 ± 22.86	79.16 ± 24.12	80.79 ± 28.97	93.29 ± 52.38	< 0.001
Hypertension, no. (%)	168 (60.00)	126 (73.68)	62 (71.26)	99 (85.34)	< 0.001
Systolic Pressure (mmHg)	139.88 ± 19.20	145.32 ± 23.45	144.14 ± 21.11	150.97 ± 25.79	< 0.001
Diastolic Pressure (mmHg)	83.47 ± 12.56	83.50 ± 11.98	80.66 ± 12.53	81.59 ± 12.17	0.165
Pulse Pressure (mmHg)	56.40 ± 14.71	61.82 ± 20.16	63.48 ± 16.31	69.37 ± 20.43	< 0.001

^a^Plus-minus values are means±SD. Percentages do not sum to 100 because of rounding

*BMI* Body mass index, *CAD* Coronary artery disease, *Cr* Creatinine

### Association between brachial PP and CAD

Both univariate and multivariate analysis were used to analyze the relationship between the brachial PP and the number of diseased vessels. Increasing brachial PP (per 1 mmHg) were associated with the increased number of diseased vessels (β = 0.01, SE = 0.00, *P* < 0.0001), after adjusted for age, gender, BMI, smoking, diabetes, hypercholesterolemia and Cr. This association was also observed when patients divided into non-hypertension and hypertension (Table 2).

**Table 2:** Association of brachial PP with the increased number of diseased vessels

Variables	n	Mean ± SD (mmHg)	Crude			Adjusted		
			β	SE	*P*	β	SE	*P*
Brachial PP (per 1 mmHg)	654	61.06 ± 18.15	0.01	0.00	< 0.0001	0.01	0.00	< 0.0001
Non-Hypertension	199	52.27 ± 14.37	0.01	0.00	0.004	0.01	0.00	0.029
Hypertension	455	64.91 ± 18.30	0.01	0.00	< 0.0001	0.01	0.00	< 0.0001

Adjusted for: Age; Gender; BMI; Smoking; Diabetes; Hypercholesterolemia; Cr

Of the 654 symptomatic patients, 203 (31.04%) had multivessel CAD (luminal stenosis ≥50% in two or more vessels). Among the included patients, the risk of multivessel CAD increased significantly in patients with brachial PP ≥ 60 mmHg (OR = 1.69, 95% CI = 1.14–2.48, *P* = 0.0084) and as per 1 mmHg increased in brachial PP (OR = 1.02, 95% CI = 1.01–1.03, *P* = 0.0002), independent of age, gender, BMI, smoking, diabetes, hypercholesterolemia and Cr (Table 3). In subgroup analysis, the increased adjusted risk of multivessel CAD was observed as per 1 mmHg increased in brachial PP, measured at non-hypertension group (OR = 1.04, 95% CI = 1.01–1.07, *P* = 0.0135) and hypertension group (OR = 1.02, 95% CI = 1.01–1.03, *P* = 0.0037), while a trend for patients with brachial PP ≥ 60 mmHg, among non-hypertension group (OR = 2.71, 95% CI = 0.94–5.03, *P* = 0.0706) and hypertension group (OR = 1.51, 95% CI = 0.97–2.35, *P* = 0.0660) (Table 4). Further dividing the hypertension group into isolated systolic hypertension and other types of hypertension, the risk of multivessel CAD were respectively 1.02(1.00, 1.06) and 1.02(1.01, 1.03) as per 1 mmHg increased in brachial PP. In addition, the association between increasing brachial PP and the increased risk of multivessel CAD was also observed in the subgroup of diabetes (Table 4).

**Table 3:** Association of brachial PP with Multivessel CAD

Varibales	n	Mean ± SD (mmHg)	Crude			Adjusted		
			OR	95%CI	*P* value	OR	95%CI	*P* value
PP < 60 mmHg	322	46.98 ± 7.71	1.00			1.00		
PP ≥ 60 mmHg	332	74.71 ± 14.57	1.81	(1.27, 2.58)	0.0010	1.69	(1.14, 2.48)	0.0084
PP (per 1 mmHg)	654	61.06 ± 18.15	1.02	(1.01, 1.03)	< 0.0001	1.02	(1.01, 1.03)	0.0002

Adjusted for: Age; Gender; BMI; Smoking; Diabetes; Hypercholesterolemia; Cr; Multivessel CAD was defined as luminal stenosis≥50% in two or more vessels

**Table 4:** Subgroup analysis of association between brachial PP and Multivessel CAD

Subgroups	n	OR	95%CI	*P* value
**Hypertension**				
**No**				
PP < 60 mmHg	142	1.00		
PP ≥ 60 mmHg	57	2.59	(1.27, 5.26)	0.0086
PP (per 1 mmHg)	199	1.04	(1.01, 1.06)	0.0017
**Yes**				
**All**				
PP < 60 mmHg	180	1.00		
PP ≥ 60 mmHg	275	1.62	(1.08, 2.42)	0.0195
PP (per 1 mmHg)	455	1.02	(1.01, 1.03)	0.0007
**Isolated systolic hypertension**				
PP (per 1 mmHg)	111	1.02	(1.00, 1.06)	0.0089
**Other types of hypertension**				
PP < 60 mmHg	180	1.00		
PP ≥ 60 mmHg	164	1.44	(1.03, 3.17)	0.0279
PP (per 1 mmHg)	344	1.02	(1.01, 1.03)	0.0010
**Diabetes**				
**No**				
PP < 60 mmHg	336	1.00		
PP ≥ 60 mmHg	165	1.78	(1.07, 2.49)	0.0058
PP (per 1 mmHg)	501	1.02	(1.01, 1.03)	0.0006
**Yes**				
PP < 60 mmHg	64	1.00		
PP ≥ 60 mmHg	89	1.87	(1.15, 4.43)	0.0134
PP (per 1 mmHg)	153	1.03	(1.01, 1.07)	0.0015

Multivessel CAD was defined as luminal stenosis≥50% in two or more vessels

## Discussion

In this cross-sectional study, we explored the association between brachial PP and CAD in patients with stable angina. What we found was that increasing brachial PP was significantly and independently associated with increased risk of multivessel coronary disease. This association was more pronounced in hypertension group than in non-hypertension one.

Many studies have so far investigated the association between PP and CAD. Lee et al. [16] were the first to explore the association between high PP and the presence of CAD. PP was measured both by non-invasive sphygmomanometer and invasive catheterization before surgical intervention in 159 patients of mitral valve stenosis. PP was considered to be an independent predictor of CAD, although the contribution of age, gender and mean BP was profound. They reported an accuracy of 62% in having significant CAD in the presence of a wide PP. Millar et al. [17] performed a retrospective study of the MRC Mild Hypertension Trial. They concluded that PP was a strong risk factor for coronary events in untreated hypertensive male subjects. What’ more, a study by Pařenica et al. [13] of 1075 consecutive stable male patients showed that increased aortic PP was independently associated with more severe atherosclerosis as assessed by the significant number of diseased coronary vessel.

Studies on the relationship between aortic PP and CAD are numerous, but few are directly examining brachial PP and CAD. Brachial PP, calculated by subtracting DBP from SBP, can be easily acquired without invasive equipment. As an easily available and non-invasive indicator, it can be better applied in clinical practice. Gatzka et al. [18] found that brachial PP was higher in patients with CAD than those without, which was consistent with our results. However, they only recruited 55 patients. Kim et al. [19] did a cross-sectional study of a register database, the Korean Women’s Chest Pain Registry. They similarly found a higher level of brachial PP in patients with obstructive CAD than in those without. They went a step further by focusing on gender differences in the relationship between brachial PP and the extent of CAD. In our cross-sectional study, with a certain sample size, we further explored the relationship between brachial PP and CAD in the subgroups with or without hypertension. The association was pronounced in both groups. The statistical significance, however, is greater in hypertension group.

PP, either measured in aortic or brachial artery, has shown a significant association with CAD. However, the jury is still out on the underlying mechanisms of the association between PP and CAD. In terms of hemodynamics, PP is largely determined by arterial stiffness, stroke volume and wave reflections [3]. Among them, arterial stiffness plays the most important role in the effects of an increased PP on the risk of CAD. Arterial stiffness reduces vascular compliance, causing increased SBP and decreased DBP. Increased SBP augments the cardiac load and oxygen consumption, while decreased DBP diminishes coronary perfusion leading to myocardial ischemia. Also, PP has been reported to be related to the endothelial vasomotor dysfunction in the conduit and resistance vessels in the coronary circulation [20]. These above changes in the coronary circulation may exacerbate the progression of CAD, which helps to explain the mechanisms of the association between PP and CAD. More research is needed to further confirm the mechanisms.

Although brachial PP has been reported to be associated with the risk of CAD, studies have also shown that brachial PP gives less indications on the severity of coronary atherosclerosis than aortic PP. However, as a non-invasive indicator, brachial PP can be easily acquired without invasive equipment, thus, can be more acceptable to patients and better applied in daily clinical practice. Brachial PP can not only help to identify high-risk CAD patients for early intervention, but also be applied to develop effective therapeutic strategies for these patients.

### Study limitations

The limitations of our study are worth noting. First and foremost, the design of the study was cross-sectional. However, whether there is a real causal relationship between brachial PP and CAD has not been confirmed. Secondly, the patients enrolled in our study were from a single center. The study subjects might not represent the whole population, affecting the generalization of the conclusion. What’s more, the bias in data collection should not be ignored. The treatment of antihypertensive medications within 12 months was self-reported. And the definition of hypertension was depended on previous diagnoses and the two BP measurements taken during the study. Also, the bias regarding blood pressure measurements should not be ignored.

## Conclusion

A positive relationship between brachial PP and the number of diseased coronary vessels was found in our study. Increasing brachial PP was significantly and independently associated with increased risk of multivessel coronary disease in the stable angina patients. The association was more pronounced in hypertension group than in non-hypertension one.

## Data Availability

Data and materials are available from the corresponding author upon reasonable request.

## Abbreviations

CAD: Coronary artery disease
PP: Pulse pressure
SBP: Systolic blood pressure
DBP: Diastolic blood pressure
CAG: Coronary angiography
BP: Blood pressure
LVEF: Left ventricular ejection fraction
BMI: Body mass index
Cr: Creatinine
SD: Standard deviation
SE: Standard error
OR: Odds ratio
CI: Confidence interval
